# Comprehensive Review on Phytochemicals, Pharmacological and Clinical Potentials of *Gymnema sylvestre*

**DOI:** 10.3389/fphar.2019.01223

**Published:** 2019-10-29

**Authors:** Farzana Khan, Md. Moklesur Rahman Sarker, Long Chiau Ming, Isa Naina Mohamed, Chao Zhao, Bassem Y. Sheikh, Hiew Fei Tsong, Mohammad A. Rashid

**Affiliations:** ^1^Department of Pharmacy, State University of Bangladesh, Dhaka, Bangladesh; ^2^Pharmacology and Toxicology Research Division, Health Med Science Research Ltd., Dhaka, Bangladesh; ^3^PAPRSB Institute of Health Sciences, Universiti Brunei Darussalam, Bandar Seri Begawan, Brunei; ^4^Division of Pharmacy, School of Medicine, College of Health and Medicine, University of Tasmania, Hobart, TAS, Australia; ^5^Department of Pharmacology, Faculty of Medicine, Universiti Kebangsaan Malaysia (The National University of Malaysia), Cheras, Malaysia; ^6^College of Food Science, Fujian Agriculture and Forestry University, Fuzhou, China; ^7^Faculty of Medicine, Taibah University, Almadinah Almunawwarah, Saudi Arabia; ^8^Alpro Pharmacy and Powerlife, Port Dickson, Malaysia; ^9^Phytochemical Research Laboratory, Department of Pharmaceutical Chemistry, University of Dhaka, Dhaka, Bangladesh

**Keywords:** *Gymnema sylvestre*, phytomedicine, antidiabetic, herbal medicine, traditional medicine, complementary and alternative medicine, immunomodulating, lipid lowering

## Abstract

*Gymnema sylvestre* is a plant included in Apocynaceae family and is located in many regions of Asia, Africa and Australia. This plant is widely used as a traditional therapy for different purposes. Even now it is being used as a dietary supplement due to its numerous therapeutic uses. It is known to have blood glucose lowering potential and, thus, is widely used in traditional and Ayurvedic systems of medicine. It renders glucose lowering activity due to the presence of phytochemicals, such as gurmarin, gymnemic acid as well as gymnemasaponins. Gymnema sylvestre is also known to have anti-oxidant, antibiotic, anti-inflammatory, antiviral, gastro and hepatoprotective, anticancer and lipid-lowering activities. This review discusses in details on different pharmacological and clinical potentials of Gymnema sylvestre and its chemical constituents associated with its therapeutic potentials.

## Introduction

Plants are a great concern for drug discovery exploration and a major source of our modern medicine. About 25% of modern medicines are derived from a plant source and merely 5-15% of plants have been investigated for their medicinal use (Gurnani et al., 2014). Nowadays, natural plants, herbal medicines, phytomedicines, and functional foods are extensively studied by scientists all over the world which resulted with the lucrative therapeutic potentials such as antidiabetic (Sarker, 2015; Shah et al., 2016; Rouhi et al., 2017; Chen et al., 2018), anticancer (Sheikh et al., 2017a; Sheikh et al., 2017b), immunomodulating (Goto et al., 2010; Sarker et al., 2011; Sarker et al., 2012a; Sarker et al., 2012b; Sarker and Gohda, 2013), antiobesity and lipid lowering (Kazemipoor et al., 2015; Sarker, 2015), anti-inflammatory (Imam et al., 2013) and anti-bacterial (Yasmin et al., 2009) activies. Among the potential medicinal plants, *Gymnema sylvestre*, belongs to the family of Apocynaceae, and is traditionally used for the treatment of various dieseases. It is a wild herb found in India, Africa, Australia, and China (Christopoulos et al., 2010). It is known as Meshashringi, Merasingi, Kavali, Kalikardori, Vakundi, Dhuleti, Mardashingi, Podapatri, Adigam, Cherukurinja, Sannagerasehambu, Chigengteng or Australian Cowplant, Waldschlinge in German, Periploca of the woods in English (Kanetkar et al., 2007). This plant is also recognized as ‘Gurmur’, due to having sugar lowering property (Tiwari et al., 2014). *Gymnema sylvestre* was considered as one of the major botanicals to treat diabetes in the Ayurvedic system of medicine and also is included in Indian Pharmacopoeia as an anti-diabetic plant (Singh et al., 2008). As it is useful against major diseases such as cardiovascular diseases, asthma, cancer, diabetes and obesity, different formulation of this plant is found in a number of preparations such as tea bags, health tablets, and food supplements. In various studies, *Gymnema sylvestre* is reported to be effective against arthritis, diuretic, anemia, osteoporosis, hypercholesterolemia, cardiopathy, asthma, constipation, microbial infections, indigestion, and as an anti-inflammatory agent (Tiwari et al., 2014). Although this plant has been proven valuable through its numerous useful properties, not many studies especially clinical studies on this plant are available. We aim to extensively review the therapeutic potential and phytochemical compounds present in this plant based on the published reports so far.

## Search Strategy and Terms Used

A comprehensive, electronic search was conducted for studies published before April 2019 using PubMed, SCOPUS, Web of Science, EMBASE, Elsevier, ScienceDirect, Researchgate, Google, and Google Scholar databases. Keywords related to, `Pharmacology’, ‘Antioxidant’, ‘Anti-diabetic’, ‘Anticancer’, ‘Immunomodulatory’, ‘Anti-arthritis’, Weight loss’, ‘Lipid lowering’, ‘Antimicrobial’, ‘Anti-inflammatory’, ‘Hepatoprotective’, ‘Gastroprotective’, ‘Traditional’, ‘Phytochemicals’ combined with ‘*Gymnema sylvestre*’ were used.

## Botanical Description and Taxonomy of *Gymnema Sylvestre*

*Gymnema sylvestre* (Retz.) R.Br. ex Sm. is a vulnerable and slow growing species. It appears as highly branched, woody and can climb up to the top of the tree that grows in the dry forests of central and southern India and in other regions of Asia (Wu et al., 2012; Kapoor, 2017). This is a shrub of pubescent type which has young stems and branches (Kanetkar et al., 2007). Its root system is of tap root type (Najafi and Deokule, 2011). Stems are cylindrical, branched, hard, twining, internodes terete, 0.7-17.2 cm long and 2 -10 mm in diameter (Najafi and Deokule, 2011; Pramanick, 2016). The leaves have distichous phyllotactic opposite arrangement pattern, are 2.5–6 cm long, usually ovate or elliptical and simple (Kanetkar et al., 2007). Leaves are acute or shortly acuminate, have petioles of 1- to 2-cm long, are smooth above, with a rounded base, a densely velvety pubescent beneath, and ciliate along margins, especially on the nerves. Venation is of transverse and reticulate type with a marginal vein (Kirtikar and Basu, 1975; Pramanick, 2016). Seeds are 1.3 cm long, flat with a thin marginal wing and narrowly ovoid-oblong (Chopra et al., 2002; Kirtikar and Basu, 1975). Flowers are small and yellow in color, in axillary and lateral umbel in cymes. Follicles are terete, lanceolate and of up to 3 inches in length (Kanetkar et al., 2007). Calyx is 5-lobed, ovate, obtuse, ciliated where corolla is campanulated, yellow, 5-lobed (Pramanick, 2016). Flowering of the plant occurs during August to March. Propagation through seed is difficult due to a low viability of seeds and, thus, plantation of root cuttings in June and July or plantation of terminal cuttings in February and March is done as an alternative approach (Kirtikar and Basu, 1975).

Gymnema sylvestre (Retz.) R.Br. ex Sm. is from *Gymnema* genus which belongs to *Apocynaceae* family. This genus has 49 other approved species which includes *Gymnema* acuminatum *Wall., Gymnema* brevifolium *Benth., Gymnema* chalmersii *Schltr., Gymnema* hirsutum Wight and Arn. etc. (The Plant List, 2013). The Taxonomy of the plant is presented in **Table 1**.

**Table 1:** Taxonomy of *Gymnema sylvestre* (Kirtikar and Basu, 1987).

Kingdom	Plantae
Subkingdom	Tracheobionta
Superdivision	Spermatophyta
Division	Magnoliophyta
Class	Magnoliopsida
Subclass	Asteridae
Order	Gentianales
Family	*Apocynaceae*
Sub-family	*Asclepiadaceae*
Genus	*Gymnema*
Species	*Gymnema sylvestre* (Retz.) R.Br. ex Sm.

## Traditional Uses

*Gymnema sylvestre* is mentioned in Shushruta, an ancient book on medicine as a remedy for glycosuria and urinary disorder (Nadkarni, 1986). It is a therapeutic herb having multiple potentials as mentioned in folk medicine, Ayurveda, and Homeopathic systems of medicine (Kanetkar et al., 2007). Traditionally, it has been used to treat diabetes, malaria and snake bites as well as to treat diseases caused by phlegm and piles in the Ayurvedic system of medicine (Kirtikar and Basu, 1975; Singh et al., 2008). In Ayurveda, the plant is prescribed for the treatment of dyspepsia, constipation, jaundice, hemorrhoids, renal and vesicle calculi, cardiopathy, asthma, bronchitis, amenorrhea, and leucoderma (Sastry, 1994; Nadkarni, 1996; Anis et al., 2000; Mathew, 2004). Furthermore, different parts of the plant such as the roots, stem, and leaves have been used as cardiotonic, digestive, diuretic, laxative, stimulant, stomachic, and uterine tonic in traditional medicine systems (Mathew, 2004). Various parts of this plant are used by different tribes in India such as the Sahariya tribe of Madhya Pradesh, Junglee Irulas of Nilgiri hills, Kol tribe of Chhattisgarh, and the Nayaks of Karnataka, to treat mainly asthma, eye and gastric problems, parkinsonism, urinary problems, and diabetes (Chopra et al., 2002; Anis et al., 2000; Potawale et al., 2008).

## Phytochemistry of *Gymnema Sylvestre*

Stems of *Gymnema sylvestre* were investigated using chromatographic techniques and were found to have several therapeutically important chemical compounds such as stigmasterol and triterpenoid saponin. Stigmasterol compounds have multiple therapeutic potentials including antidiabetic, hypoglycemic, antioxidant, anticancer activities. Triterpenoid saponins also exhibited anti-tumor, anti-fungal, hepatoprotective and antidiabetic potential in several studies (Matsuda et al., 1997; Kaur et al., 2011; Garai, 2014; Liu et al., 2014). Gymnemic acids and gymnemasaponins are major chemical constituents of this plant and are classified as oleanane saponins. Oleanane and dammarane type of saponins are found in the leaves of *Gymnema sylvestre* (Khramov et al., 2008). The leaves of this plant also have saponins, anthraquinones, cardiac glycosides etc. (Patel, 2017). Moreover, this plant was also observed to have tannin, quinones, flavonoids, and phenols. (Senthilkumar, 2015). The phytochemical compounds found in the analysis of *Gymnema sylvestre* is listed in **Table 2**.

**Table 2:** Phytochemical constituents of *Gymnema sylvestre*.

Constituents	Classification	Chemical Structure	References
Triterpene saponins	Gymnemic acids-acylated (tiglolyl, methylbutyroyl) derivatives of deacylgymnemic acid (DAGA) which is a 3-O-*β*-glucouronide of gymnemagenin (3*β*, 16*β*, 21*β*, 22*α*, 23, 28-hexahydroxy-Olean-12-ene).	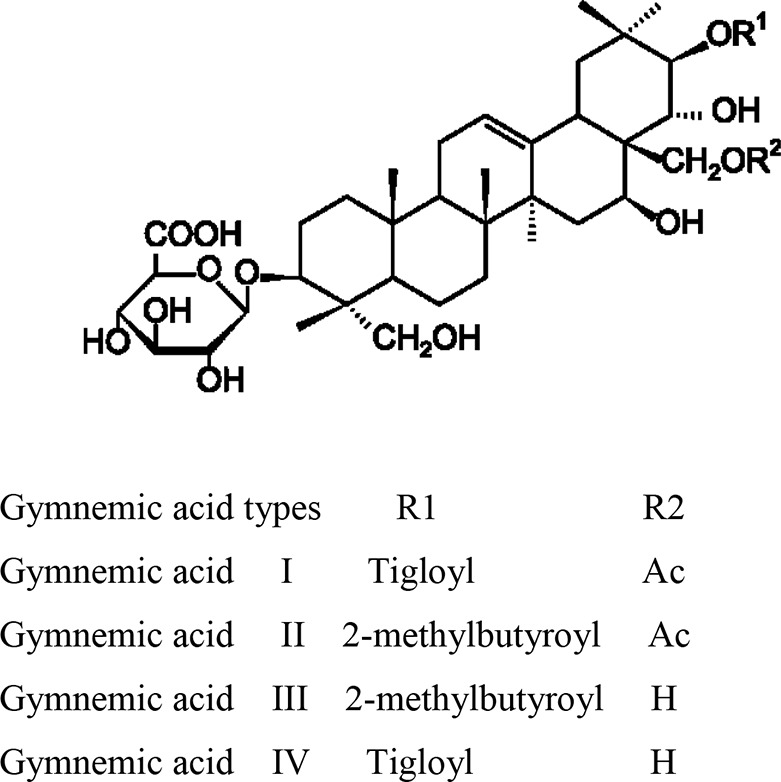	(Liu et al., 1992)
Oleanane Saponins	Gymnemic acids and gymnemasaponins	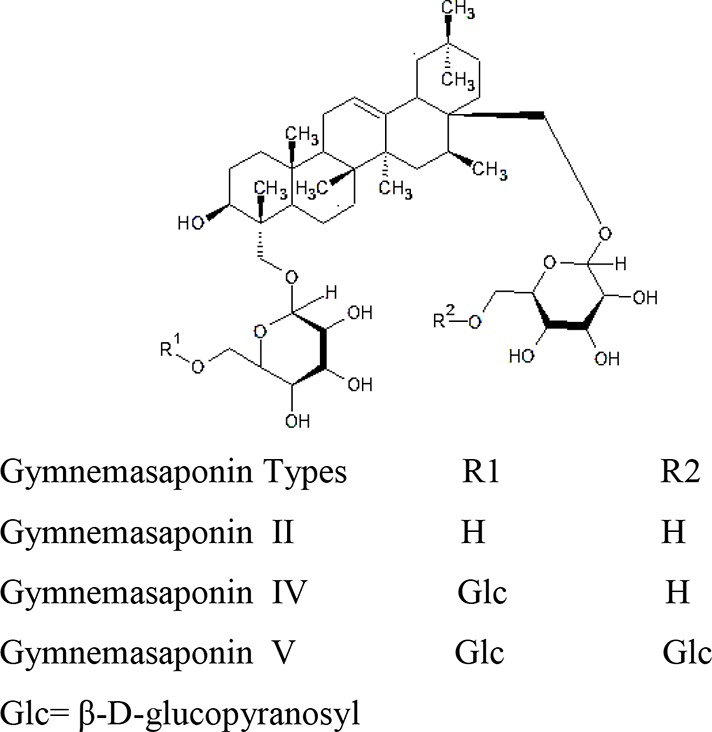	(Ye et al., 2000)
Gymnemanol	3,*β*-16,*β*-22, *α*-23-28-pentahydroxyolean-12-ene	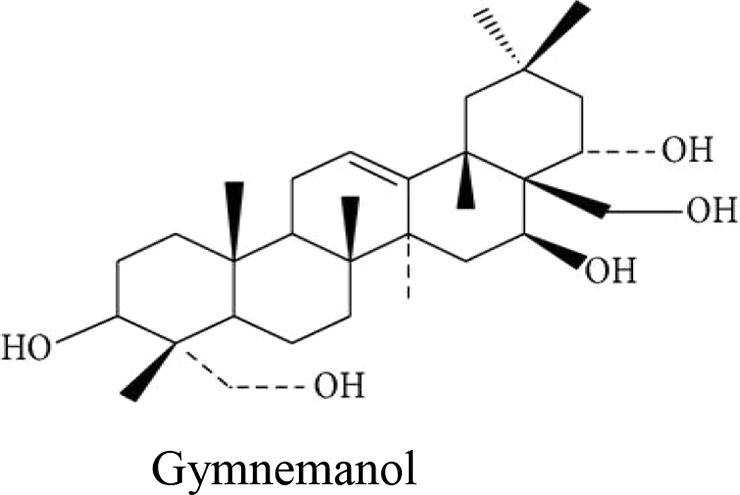	(Sahu et al., 1996)
Dammarene Saponins	Gymnemoside A, B, C,D,E,F	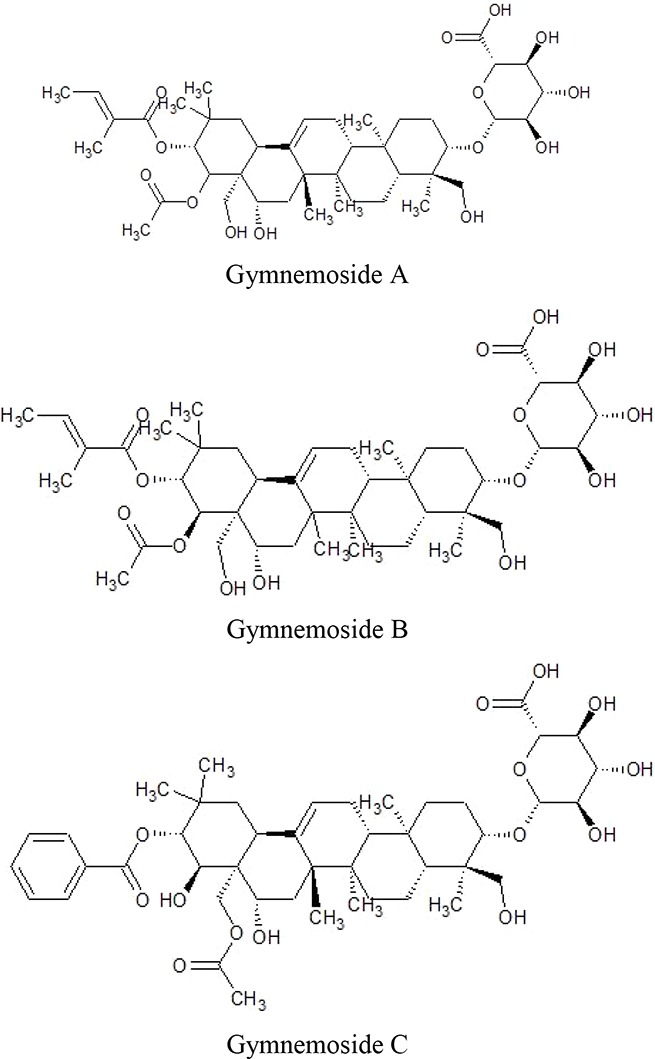 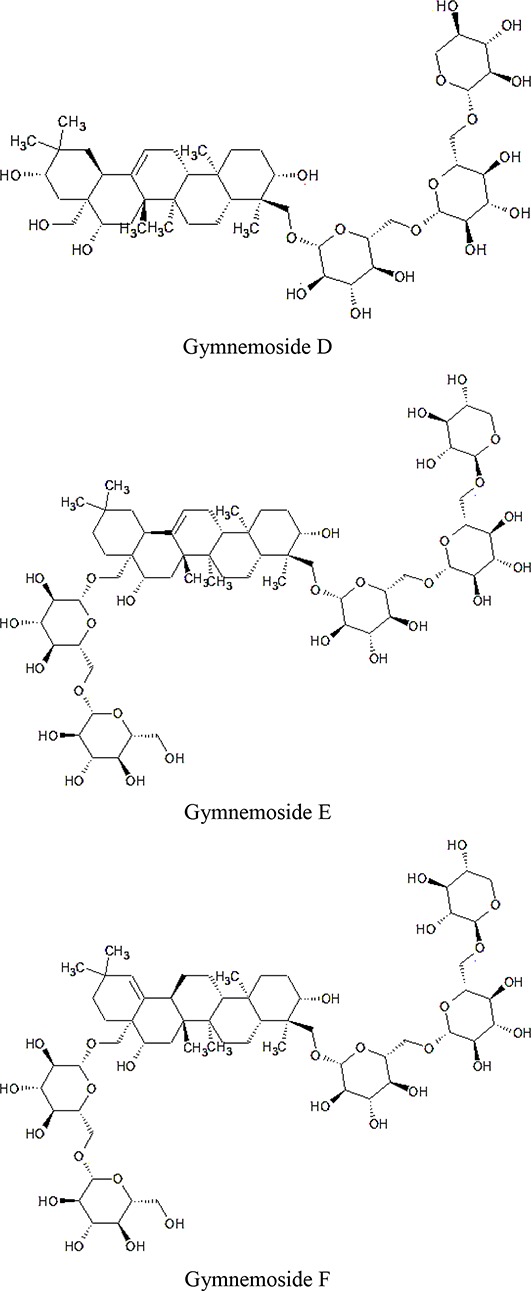	(Yoshikawa et al., 1997; Yoshikawa et al., 1998)
			
Gymmestrogenin	Pentahydroxytriterpene	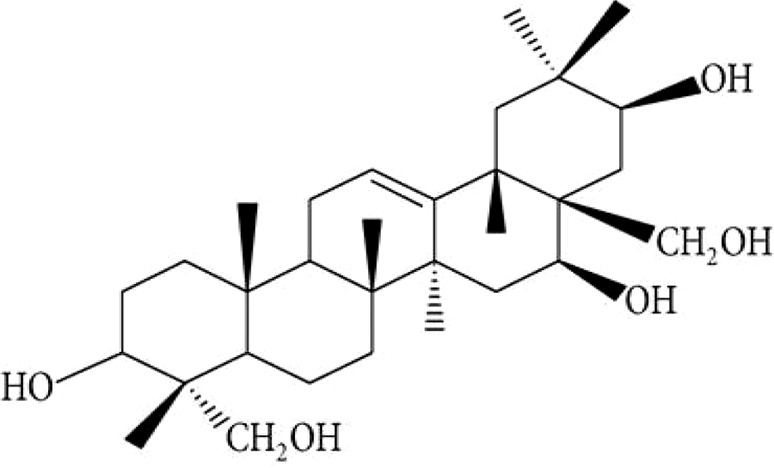	(Yoshikawa et al., 1998)
Gurmarin	A 35-Amino acid peptide with a molecular weight of 4209	<1Glu- Gln- Cys- Val- 5Lys- Lys- Asp- Glu- Leu- 10Cys- Ile- Pro-Tyr- Tyr- 15Leu- Asp- Cys- Cys- Glu- 20Pro- Leu- Glu- Cys- Lys-25Lys- Val- Asn- Trp- Trp- 30Asp- His- Lys- Cys- Ile- 35Gly>. (Glu = pyroglutamic-acid)	(Imoto et al., 1991)
Triterpenoid saponins Gymnemasin A Gymnemasin B Gymnemasin C Gymnemasin D	3-O [*β*-D-glucopyranosyl (1-3)-*β*-D-glucopyranosyl]-22-O-tiglyol gymnemanol 3-O-[*β*-D-glucopyranosyl-(1-3)-*β*-D-glucuro- nopyranosyl]- Gymnemanol 3-O-*β*-D-glucuronopyranosyl-22-O-tigloyl- gymnemanol 3-O-*β*-D-glucopyranosyl-gymnemanol	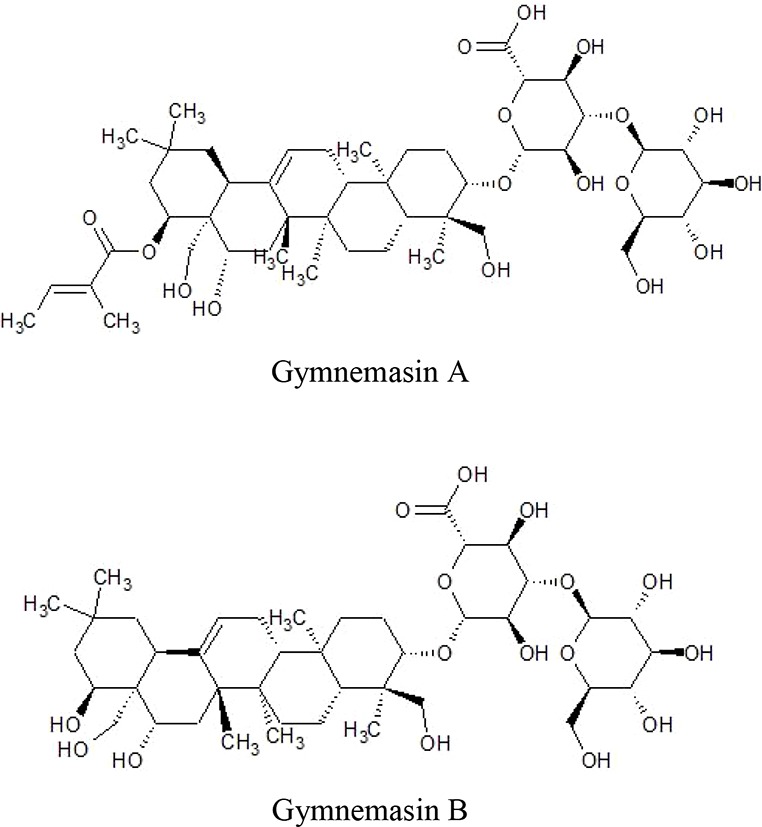 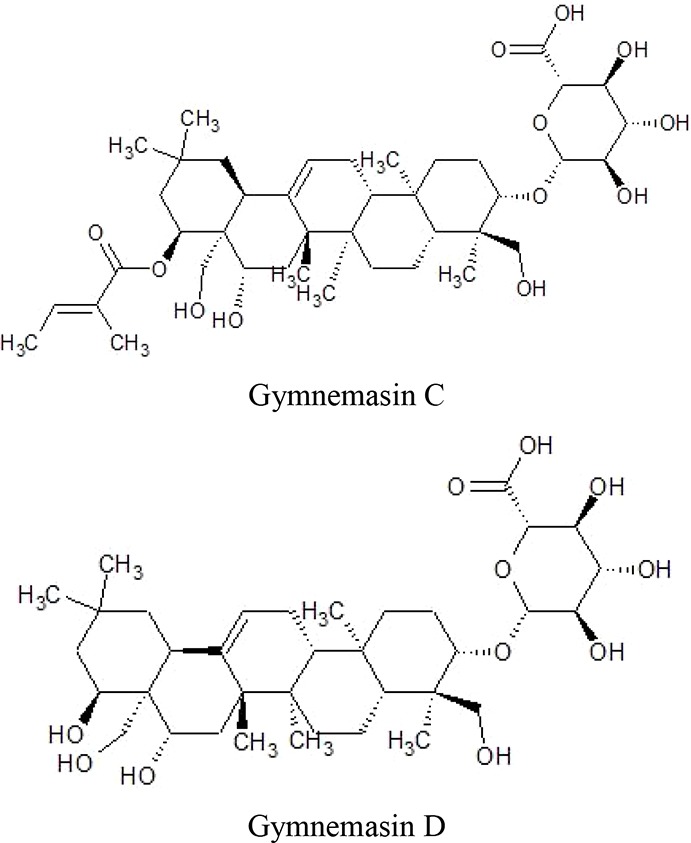	(J-GLOBAL-Japan Science and Technology Agency, n.d.a; J-GLOBAL-Japan Science and Technology Agency, n.d.b; J-GLOBAL-Japan Science and Technology Agency, n.d.c; J-GLOBAL-Japan Science and Technology Agency, n.d.d)
			
Flavonol glycoside	Kaempferol 3-O-*β*-D-glucopyranosyl-(1-4)-*α*- L-rhamnopyranosyl-(1-6)- *β*-D-galactopyranoside	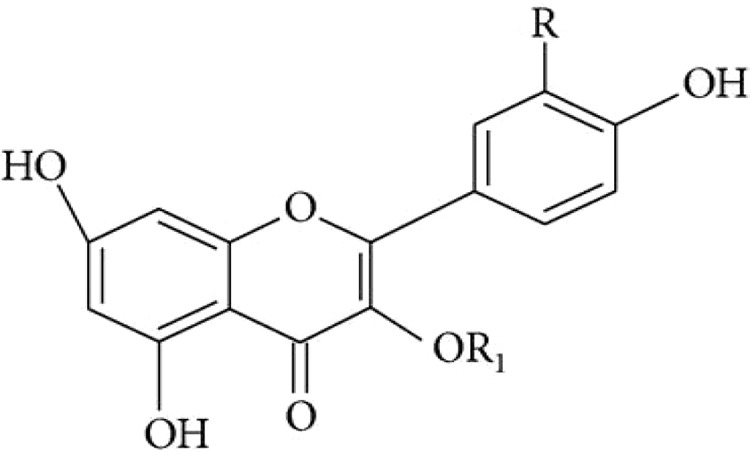	(Liu et al., 2004)
Sterols	Stigmasterol	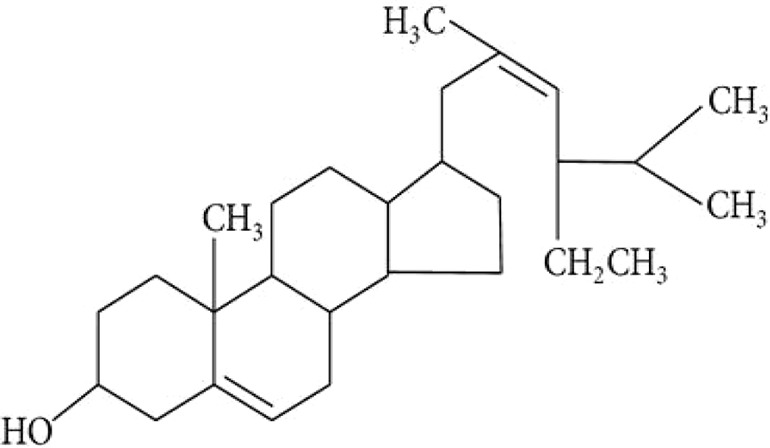	(Potawale et al., 2008)
Lupeol		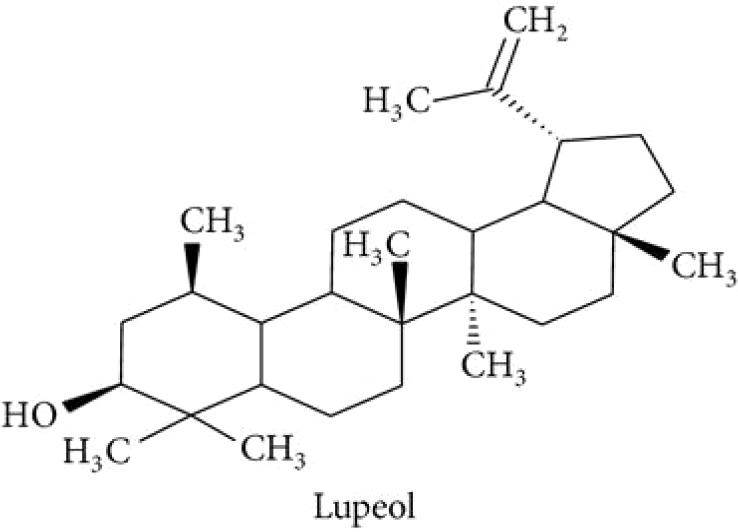	(Sinsheimer et al., 1970)
d-Quercitol		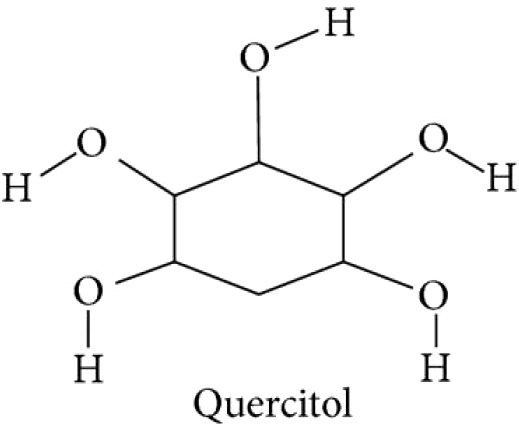	(Potawale et al., 2008)
Parabin		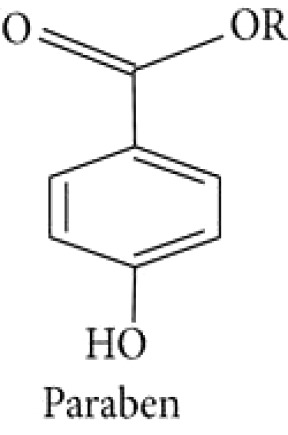	(Sinsheimer et al., 1970)
	Quercitol	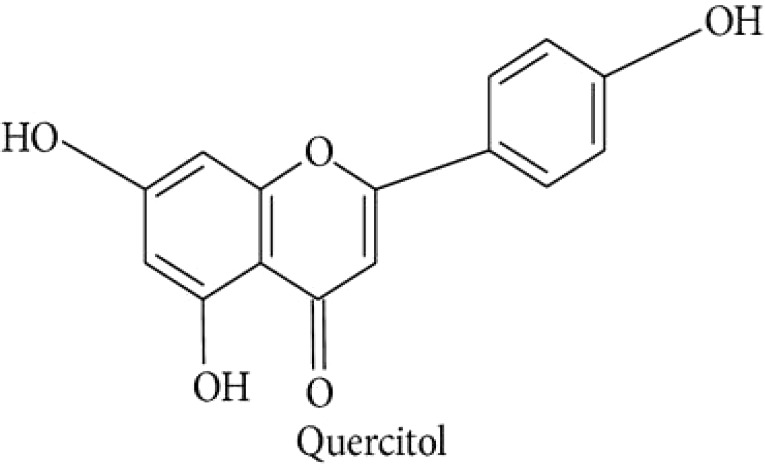	(Tiwari et al., 2014).
	Conduritol A	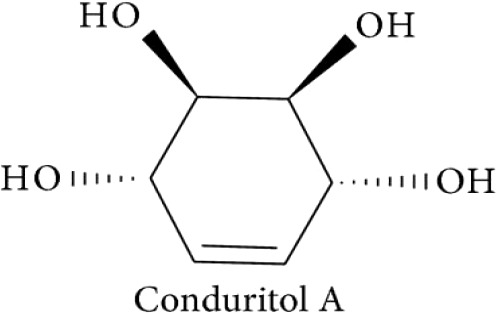	(Tiwari et al., 2014).

## *In Vitro* and *in Vivo* Pharmacological Activity Reports on *Gymnema Sylvestre*

**Figure 1:**
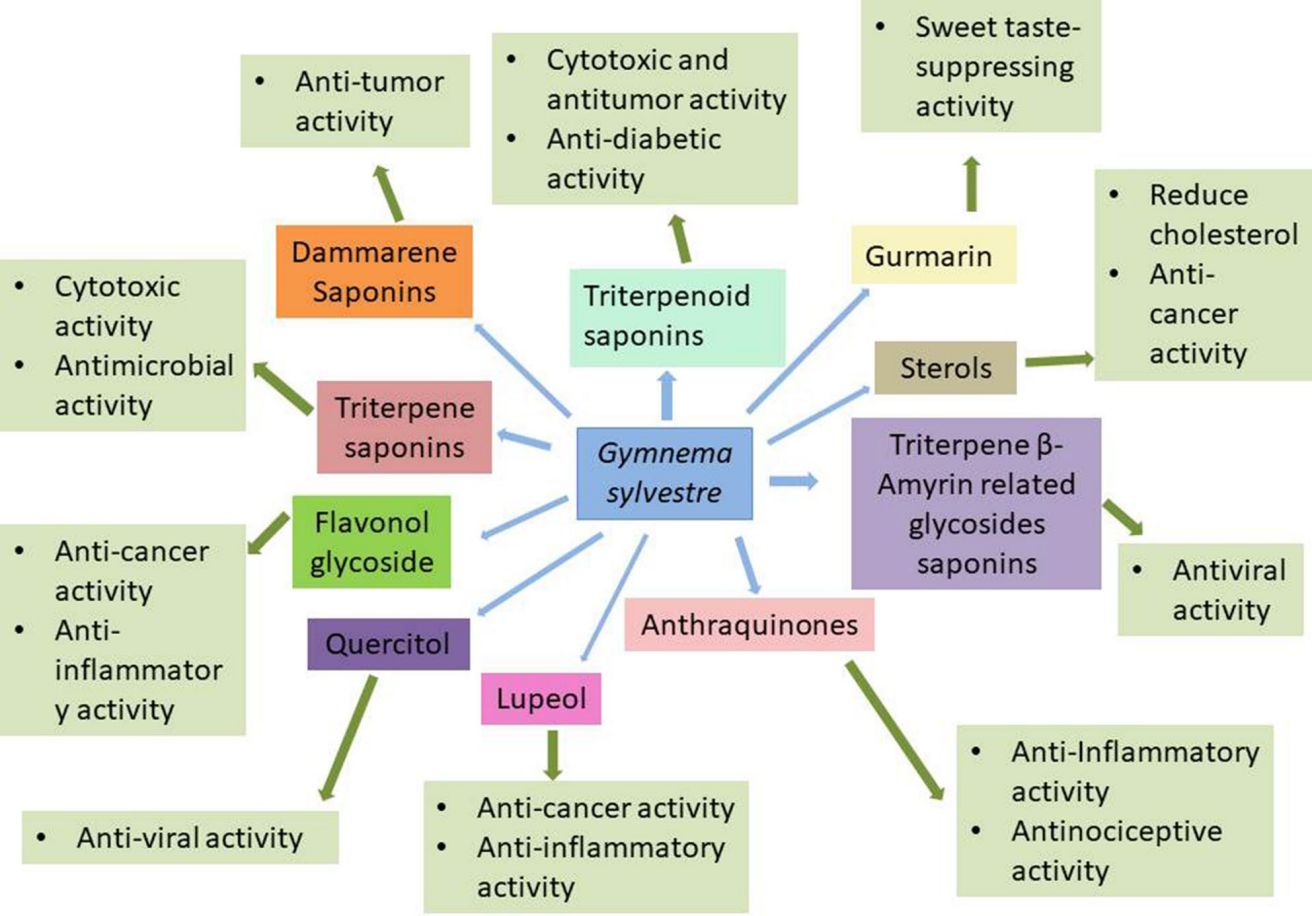
Phamacological activities of the constituents of *Gymnema sylvestre*.

*In vitro* and *In vivo* investigation of the therapeutic importance of *Gymnema sylvestre* revealed multifarious pharmacological potentials including anti-cancer, immunosuppressive, gastro-protective, hypoglycemic, anti-inflammatory, anti-infectious, and most importantly anti-diabetic activities. The pharmacological activities of phytochemicals derived from *Gymnema sylvestre* have been presented in **Figure 1** and its reports are summarized in **Table 3**.

**Table 3:** Summary of Pharmacological Effects of *Gymnema sylvestre*.

*Gymnema sylvestre* Extract	Type of study	Study Model	Activities	References
Water	*In vivo*	Alloxan induced diabetic and normal Male Wister albino rats	Blood glucose level was reduced	(Sathya et al., 2008; Ahmed et al., 2010)
	*In vitro*		• Anti- oxidant activity	(Arun et al., 2014)
	*In vivo*	Albino Rats	• Anti-allergic • Anti-stress • Inhibition of aspirin-induced gastric ulcers	
	*In vitro*	*Staphylococcus aureus and Staphylococcus epidermidis*, *Escherichia coli*, *Klebsiella pneumoniae 1, Klebsiella pneumoniae* *2, Pseudomonas aeruginosa, Salmonella typhimurium 1*, *Salmonella typhimurium 2, Shigella* *Flexneri, Candida albicans*, *Candida tropicalis*, *Methicillin-resistant Staphylococcus* *Aureus*	• Antimicrobial activity against *Pseudomonas aeruginosa, Candida albicans, Klebsiella pneumoniae 1, Pseudomonas aeruginosa, Salmonella typhimurium 2, Escherichia coli, Staphylococcus aureus, Methicillin-resistant Staphylococcus aureus* • *Enterococcus faecalis* and *Staphylococcus epidermidis* were insensitive to the plant extract.	(Arora and Sood, 2017)
Alcohol	*In vivo*	Wister Rats	• Fat digestibility was decreased • Excretion of neutral sterols and acid steroids into feces was increased • Decreased the total cholesterol and triglyceride levels in serum • The decrease in body weight • Inhibition of fat accumulation	(Shigematsu et al., 2001a; Shigematsu et al., 2001b)
Acetone	–	Culex tritaeniorhynchus Giles (Diptera: Culicidae)	• Larvicidal Activity	(Elumalai et al., 2013)
Methanol	–	Culex tritaeniorhynchus Giles (Diptera: Culicidae)	• Larvicidal Activity	(Elumalai et al., 2013)
	*In vivo* and *In vitro*	Alloxan induced diabetic Wistar rats	• Increased the weight of the whole body, liver, pancreas • Increased liver glycogen content • Regeneration of β-cells	(Ahmed et al., 2010)
	*In vitro*	L6 myotubes and 3T3 L1 murine adipocyte cell line	• Enhanced GLUT-4 and PPAR-γ gene expressions • Enhanced expression of adiponectin and leptin	(Kumar et al., 2016)
	*In vivo*	Wister rats	• Reduced blood glucose level • Reduced triglyceride, cholesterol level • Lowered urea, creatinine level	(Dholi and Raparla, 2014)
Chloroform	–	Culex *tritaeniorhynchus Giles* (Diptera: Culicidae)	• Larvicidal Activity	(Elumalai et al., 2013
	*In vitro*	*B. subtilis, S. epidermis, E. faecalis, S. aureus*, *P. aeruginosa, E. cloacae; E. aerogene; E. coli*, *S. typhimuriumand K. pneumoniae*	• Shown wide range of inhibitory activity against *Staphylococcus aureus* and *Klebsiella pneumoniae*	(Chodisetti et al., 2013)
OSA	*In vitro*	MIN6 mouse β-cell and human islets of Langerhans	• Increased insulin secretion	(Liu et al., 2009)
	*In vivo*	Insulin-resistant ob/ob mice	• Improved glucose-intolerant status	(Al-Romaiyan et al., 2010)
	*In vitro*	Isolated mouse islets	• Increased insulin secretion	
	*In vitro*	Human Islets of Langerhans	• Increase in Insulin Secretion	
Gymnemic Acids extracted from *Gymnema sylvestre*	*In vitro*	Male Hartley Guinea-pigs	• Suppressive effect on the H-65 *K*^+^ induced contraction of the ileal longitudinal muscle	(Shimizu et al., 1997)
	*In vitro*	Guinea-pigs and Wister rats	• Suppression of glucose invoked transmural potential difference increase in the inverted intestines	
	*In vivo*	Male Sprague-Dawley Strain Rats	• Suppression of Glucose level	
	*In vivo*	Male rats of the Wistar-ST strain	• Increased fecal cholesterol and CA-derived bile acid excretion	(Nakamura et al., 1999)
Ethanol Extract	*In vivo*	Ulcerative colitis induced Wister albino rats	• Suppressed oxidative and inflammatory response • Protected colonic mucosal content	(Aleisa et al., 2014)
	*In vivo*	Streptozotocin-induced diabetic Albino Rats	• Establishes blood glucose homeostasis Brings glycoconjugate components to near normal levels	(Shanmugasundaram et al., 1988)
	*In vitro*	Human skin melanoma cell line (A375) and normal liver cell line (WRL-68)	• Apoptotic effect on A375 cells	(Chakraborty et al., 2013)
	*In vivo*	Female ICR mice	• Showed inhibitory effects on TPA-induced inflammation	(Yasukawa et al., 2014)
	*In vivo*	Streptozotocin-induced diabetic Wistar albino rats	• Reduction in the pain threshold Reduction in body weight • Mitigation of blood glucose levels and increased insulin level • Attenuated the elevated levels of cytokines in the serum and sciatic tissues	(Fatani et al., 2015)
	*In vitro*		• exhibited strong antioxidant activity	(Kang et al., 2012)
	*In vivo*	Streptozotocin induced diabetic Sprague−Dawley male rats	• Decreased the activity of glutathione peroxidase in cytosolic liver • Reduced glutamate pyruvate transaminase in serum to normal levels	(Daisy et al., 2009)
	*In vivo*	Streptozotocin-induced diabetic Wister rats	• Showed hypoglycemic and hypolipidemic activity	
Dihydroxy gymnemic triacetate isolated from *Gymnema sylvestre*	*In vitro*	Mouse pancreatic *β*-cell lines (MIN6)	• Inhibited yeast *α*-glucosidase, sucrase, maltase, and pancreatic *α*-amylase • Increased insulin secretion • Showed protection against H_2_O_2_ induced ROS generation	(Shenoy et al., 2018)
Isolated Triterpene Glycoside fraction from *Gymnema sylvestre*	*In vitro*	Wister Rats	• Inhibited Glucose-Stimulated Gastric Inhibitory Peptide Secretion	(Fushiki et al., 1992)

### Antidiabetic Activity

The most widely known effect of *Gymnema sylvestre* is anti-diabetic activity. Ethanol extract of this plant is reported to reduce glucose level by 46% where the water extract reduced glucose level by 26% and methanol extract by 12% (Mcburney and Gent, 1978; Luo et al., 2006; Kosta and Tiwari, 2009; Shah et al., 2011; Shah et al., 2012). In dexamethasone-induced insulin resistance rats, aqueous extract of this plant was found to be improving the altered glucose, insulin and lipid profile (Kumar S, et al., 2015). Administration of this plant in a diabetic animal model resulted in reductions in the blood levels of insulin, protein, triglycerides, cholesterol, and glucose, as well as a reduction in body weight and was found to improve liver histopathology (Sujin, 2008). In another study where alloxan-induced diabetic rats were used, this plant extract significantly (*p* < 0.05) reduced fasting blood glucose level, total cholesterol, serum triglycerides and increase HDL-cholesterol level and is also described to significantly alter (*p* < 0.05) the elevated level of urea, uric acid and creatinine levels in diabetic rats to nearly normal levels (Sathya et al., 2008; Mall et al., 2009). *Gymnema sylvestre* reduced the level of blood glucose levels after both acute and chronic administration of methanolic extract of this plant on Wister rats (Dholi and Raparla, 2014). In the case of Streptozotocin-induced diabetic rats, it has been shown that treatment using this plant significantly (*p* < 0.05) decreased the elevated blood glucose, ALT, AST, triglycerides, total cholesterol, LDL-cholesterol, and malondialdehyde, and significantly (*p* < 0.05) increased insulin, HDL-cholesterol, and erythrocyte superoxide dismutase levels in diabetic rats and also is capable of regenerating insulin producing β-cells (Aralelimath and Bhise, 2012; Shafey et al., 2013; Kumar et al., 2017; Ahmed et al., 2017). Gymnemic acids (a type of triterpene saponin compounds) are the class of active constituents isolated form *Gymnema sylvestre*. It was found that gymnemic acid IV given at a dose of 3.4/13.4 mg/kg adaministered for 6 hours decreased blood glucose levels by 14.0–60.0% as compared to glibenclamide. Also, gymnemic acid IV increased plasma insulin levels in STZ-diabetic mice when administered at a concentration of 13.4 mg/kg (Sugihara et al., 2000). In a study, oral administration of small concentrations (0.2 g/kg) of this plant produced a reduction in the elevated levels of blood sugar induced by sucrose (Kang et al., 1990). However, Galletto et al. (2004) also informed an absence of anti-diabetic activity of *Gymnema sylvestre* in an alloxan treated animal model (Galletto et al., 2004).

### Mechanism of Action of *Gymnema Sylvestre* for Antidiabetic Activity

**Figure 2:**
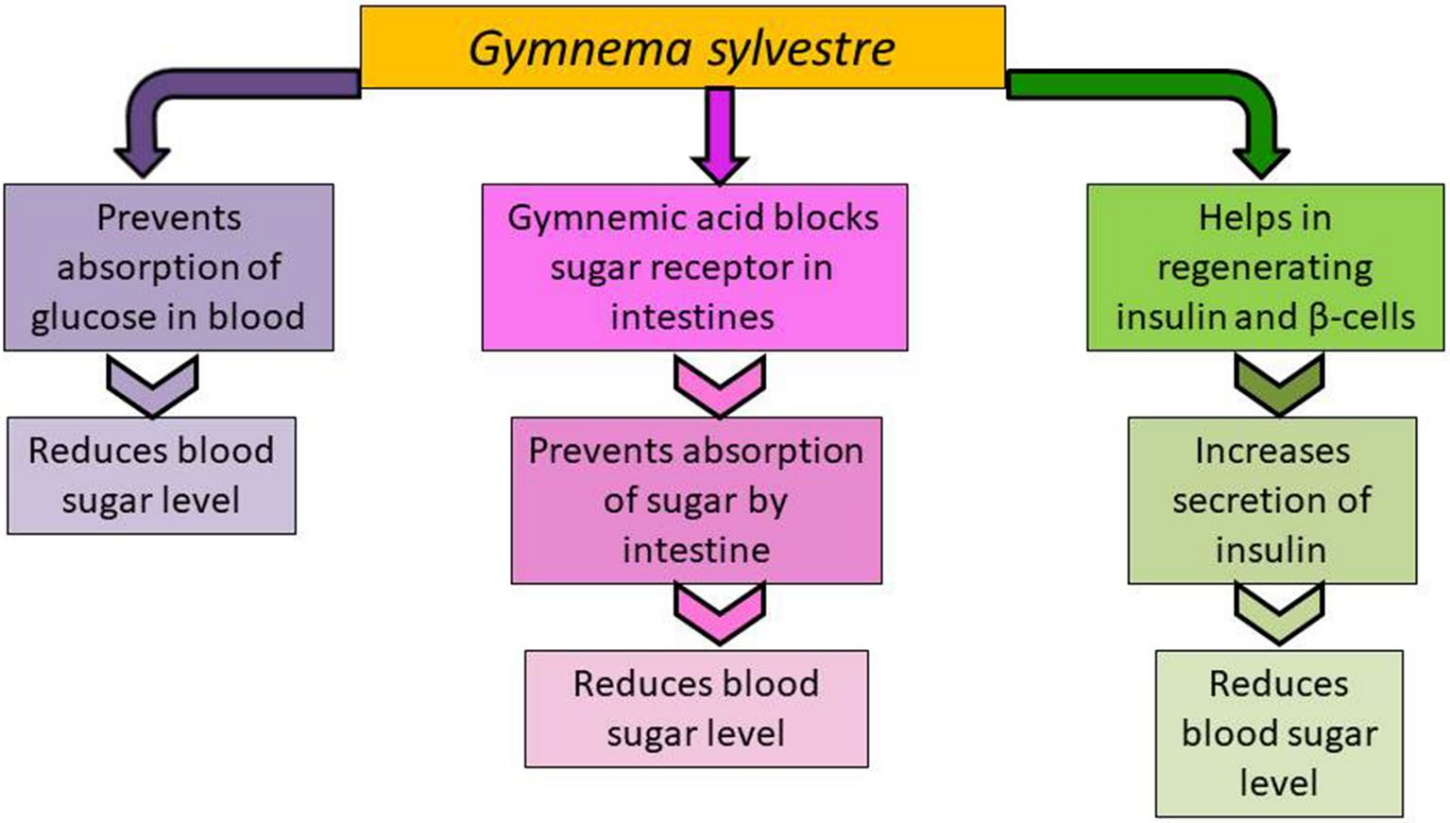
Mechanism of glucose lowering activity by *Gymnema sylvestre*.

Several mechanisms have been proposed to explain the anti-diabetic activity of *Gymnema sylvestre* (**Figure 2**). Gymnemic acids can prevent absorption of sugar molecules by the intestine, which leads to a reduction in blood sugar levels (Tiwari et al., 2014). One of the constituents of *Gymnema sylvestre* is gymnemic acid which is a mixture of saponins (Yoshikawa et al., 1993). The atomic arrangement of gymnemic acid molecules is similar to that of glucose molecules and it blocks the receptor site for sugar in the intestines, preventing the absorption of sugar which reduces blood sugar level (Sahu et al., 1996). Rapid screening by Affinity Ultrafiltration-HPLC-MS shows that it contains α-glucosidase inhibitors (Chen and Guo, 2017). It is reported to increase the activity of enzymes which are insulin dependent including hexokinase, glycogen synthetase, glyceraldehydes 3-phosphate dehydrogenase, and glucose 6-phosphate dehydrogenase, and to decrease the activity of insulin-independent enzymes such as glycogen phosphorylase, gluconeogenic enzymes, glucose 6-phosphatase, fructose 1,6- diphosphatase, and sorbitol dehydrogenase, which also increases phosphorylase activity. *Gymnema sylvestre* was also found to increase the secretion of insulin and the possible role in regenerating insulin as well as β-cell was suggested (Shanmugasundaram et al., 1990a; Nakamura et al., 1999; Aralelimath and Bhise, 2012). In a study, methanol extract of this plant showed increased effect on β-cell regeneration and was extrapolated that this plant might be able to completely recover pancreatic-cells function and thus treating type I diabetes (Ahmed et al., 2010).

### Anticancer Activity

*Gymnema sylvestre* was found to have anticancer activity in various investigations. Its constituent gymnemagenol (C30H50O4) showed positive anticancer activity against HeLa cells (Khanna, 2010). The ethanolic, ethyl and chloroform extract were tested for anticancer activity against A549 (human lung adenocarcinoma) and MCF7 (human breast carcinsoma) cell lines. Theses extract revealed anticancer activity with a similar IC50 value against MCF cell lines where in the case of A549, ethyl and chloroform extract were more active than the ethanol extract (Srikanth et al., 2010). Ethanolic extract of *Gymnema sylvestre* showed anticancer activity in A375 cells (human skin melanoma). It showed cytotoxic activity against A375 cell and antitumor activity in skin Papilloma model where, in the case of normal liver cells WRL-68, it showed no cytotoxic activity (Chakraborty et al., 2013). It was revealed to have significant (*p* < 0.0001) inhibitory effect against intestinal breast cancer resistance protein (BCRP) (Tamaki et al., 2010). In a study, the administration of flavonoids was found to be inhibiting BCRP and subsequently, improving multi-drug resistance of BCRP substrates that was induced by it (Imai et al., 2004). Thus, it can be suggested that inhibition of this protein by *Gymnema sylvestre* may improve the activity of BCRP substrates methotrexate, daunorubicin, topotecan, epirubicin, flavopiridol, and so on by increasing systemic availability and absorption (Mao, 2005). Ethanolic extract of this plant exhibited antiproliferative effects in mice with two-stage carcinogenesis with a 50% inhibitory dose of 50–555 nmol/ear (Yasukawa et al., 2014). Polysaccharides (GSP11, GSP22, GSP33, GSP44 and GSP55) isolated from *Gymnema sylvestre* was reported to have anticancer activity by improving immunological function through increasing phagocytic function, enhanced serum hemolysin levels, thymus, and spleen indexes. GSP11 and GSP33 showed inhibitory rates of 78.6% and 83.8%, respectively, against U937 cells and GSP22 showed activity against SGC cells with an inhibitory rate of 78.2%. (Wu et al., 2012). In another study, antitumor potential of this plant was observed when methanolic extract of *Gymnema sylvestre* was administered on Swiss albino mice where papillomagenesis was induced using carcinogen 7, 12 - dimethylbenz (a) anthracene (DMBA). Decreased tumor incidence, tumor burden and the cumulative number of papillomas were observed after the treatment with the plant extract (Agrawal et al., 2016).

### Lipid-Lowering Activity

*Gymnema sylvestre* leaf extract was observed to possess very potent hypolipidaemic activity. In a study, *Gymnema sylvestre* leaf extract was administered to Wister female rats. These rats were introduced to hyperlipidemia by high-fat diet. It was detected that this extract significantly lowered the level of cholesterol (*p<* 0.01), low-density lipoprotein (LDL) (*p<* 0.01), and triglyceride (*p<* 0.01) as well as increased the level of high-density lipoprotein (HDL) (*p<* 0.001) effectively (Singh et al., 2017). Furthermore, hydro-alcoholic leaf extract of *Gymnema sylvestre* was also observed to have lipid-lowering potential. In this study, rats were given high cholesterol for seven days and a higher level of cholesterol, triglyceride, LDL, and a lower level of HDL was observed. After seven days, these rats were treated with *Gymnema sylvestre* extract and it was reported to lower the elevated cholesterol, triglyceride, LDL level and increase the HDL level. It was suggested that this plant renders lipid-lowering potential due to the presence of acidic constituents such as flavonoids, saponins, tannins etc. (Rachh et al., 2010; Dholi and Raparla, 2014). Similarly, in several other studies, it was reported to reduce triglyceride, cholesterol, very low‐density lipoprotein (VLDL) and low‐density lipoprotein (LDL) in diabetic rats (Bishayee and Chatterjee, 1994; Kumar et al., 2013).

### Antimicrobial Activity

Different extracts and isolated bioactive compounds of *Gymnema sylvestre* were reported to have anti-microbial potential against several microorganisms. Methanolic extract of the leaves of this plant was reported to show antimicrobial activity against *E. coli, B. cereus, C*. albicans, and *C*. kefyr. The aqueous extract showed moderately anti-microbial activity against *S. aureus, C*. krusei, *C. perfringens* type-A and *C*. kefyr where the hexane extract showed activity against *S. aureus, B. cereus, S*. enterica, H. paragallinarum and *C. perfringens* type-A (David and Sudarsanam, 2013; Tahir et al., 2017). Both aqueous and ethanol extract is active against pathogenic Salmonella species (*Salmonella typhi, S. Typhimurium*, and *S. paratyphi*). Ethanolic, chloroform, and ethyl acetate extracts were reported to be active against *P*. vulgaris, *E. coli, P*. aeroginosa, K. pneumoniae, and *S. aureus* (Pasha et al., 2009; Paul and Jayapriya, 2009). Swami and Prabakaran (2012) observed that this plant was effective against several gram positive and negative bacteria such as *S. aureus, E. Coli, K. pneumoniae* and *P. aeruginosa*. In a study where antimicrobial activity was measured using a disk diffusion method, gymnemic acid, isolated from this plant, also showed antimicrobial activity against *E. coli, V. cholera, S. mutans, S. aureus, A. niger* and *C. albicans* with zone of inhibition of 8.65 mm, 6.00 mm, 7.12 mm, 9.25 mm, 6.43 mm and 8.60 mm, respectively (Gupta and Singh, 2014). It has anti-microbial potential against a wide range of microorganisms including *E. coli, P. aeruginosa, B. subtilis, E. hirae, M. luteus, S. aureus* and *C. albicans* (Thanwar et al., 2016; Arora and Sood, 2017; Gunasekaran et al., 2019). Antibacterial activity of gymnemic acid, a triterpene saponin, isolated from *Gymnema sylvestre* was also studied against *E. coli* and *B. cereus* and it was found active against the microbes (Shivanna and Raveesha, 2009). Recently, antimicrobial properties of *Gymnema sylvestre* leaf extract have been enhanced using poly-ε-caprolactone nanofibers (Ramalingam et al., 2019) or by using ZnO nanoparticles (Karthikeyan et al., 2019). *Gymnema sylvestre* with ZnO nanoparticles was found to be effective against gram positive *Staphylococcus aureus* and *Streptococcus pneumoniae* bacteria and gram negative *Klebsiella pneumoniae, Shigella dysenteriae, Escherichia coli, Pseudomonas aeruginosa* and *Proteus vulgaris* bacterial strains (Karthikeyan et al., 2019). Using poly-ε-caprolactone nanofibers with this plant was potently active against methicillin-resistant *Staphylococcus aureus, Staphylococcus aureus, Pseudomonas aeruginosa, Staphylococcus epidermidis and Escherichia coli* (Ramalingam et al., 2019).

### Antioxidant Activity

Ethanol extract of this plant revealed significant (*p* < 0.05) 1,1-diphenyl-2-picrylhydrazyl (DPPH) radical scavenging activity and showed better antioxidant potential than *A. bilimbi* and *C. frutescens* (Rahman et al., 2014). Anti-oxidant activity of *Gymnema sylvestre* against DPPH was also observed in an investigation by Rupanar et al. (2012). This plant was found to have better DPPH radical scavenging than butylated hydroxyl toluene (BHT) and in another study it was also found to reduce LDL oxidation (Ohmori et al., 2005; Rupanar et al., 2012). Recently, in another study, hydroxyl free radical scavenging activity and significant antioxidative potential of this plant against DPPH was reported where DPPH inhibition was at the level of 87.3% and hydroxyl free radical inhibition was 59.8% (Gunasekaran et al., 2019). It was also found to have significant radical scavenging activity against ferric (*p* < 0.05), super oxide (*p* < 0.05) and also against hydrogen peroxide (*p* < 0.05) (Rachh et al., 2009). *Gymnema sylvestre* showed antioxidant activity in several conditions such as against high fat diets, hydrogen peroxide, nitric oxide, and superoxide radically induced oxidative stress in rats (Arun et al., 2014; Kishore and Singh, 2015; Chakrapani and Periandavan, 2018).

### Antiarthritic Activity

Aqueous and petroleum extract of *Gymnema sylvestre* revealed significant (*p* < 0.01) antiarthritic activity (Malik et al., 2010). It was suggested that *Gymnema sylvestre* may have reduced the release of inflammatory mediators which is necessary to reduce bone destruction in anti-arthritic condition (Malik et al., 2010). In another study, ethanolic extract of the root of *Gymnema sylvestre* reduced carrageenan rat paw oedema significantly (*p* < 0.01) and inhibited 39-75% of histamine induced rat paw oedema (Shankar and Rao, 2008)

### Immunomodulating Activity

Methanolic leaf extract of *Gymnema sylvestre* (MLEGS) showed immunosuppressive activity in Swiss Albino mice when it was tested by performing hemagglutination antibody (HA) titer, delayed-type hypersensitivity (DTH) tests and flow cytometric techniques for the estimation of B lymphocytes (CD3 and CD19) and Th2 cytokines (IL-2, IFN-γ and IL-4). This plant significantly reduced primary and secondary antibody response and DTH response in a dose-related manner. At 200 mg/kg body weight, the maximal reductions occurred in the production of CD3, CD19, IL-2, IFN-γ and IL-4 at the level of 31.59, 32.12, 29.51, 32.45 and 33.53%, respectively (Ahirwal et al., 2015). However, it was also perceived that *Gymnema sylvestre* enhances the level of myeloid and lymphoid components of the immune system. Methanolic extract of this plant significantly increased (*p* < 0.05) the stimulation of Nitric oxide (NO) and Reactive Oxygen Species (ROS) by stimulation of macrophage activity and, also, significantly (*p* < 0.05) reduced nitroblue tetrazolium (Singh et al., 2015). Aqueous extract of *Gymnema sylvestre* also stimulated the phagocytic function of human neutrophils suggesting an immunostimulatory activity (Jitender et al., 2009). Ethanol extract of this plant was observed to improve immunosuppressed condition induced by cyclophosphamide in Albino Rats. In this study, the plant extract significantly improved haemagglutination titer, phagocytic activity and decreased paw edema (*p* < 0.01, *p* < 0.05 and *p* < 0.05 respectively), when compared with cyclophosphamide treated control (Kar et al., 2019). In another study, potent immunostimulatory potential of the aqueous extract of this plant was observed (Gupta et al., 2009).

### Anti-Inflammatory Activity

Methanoic extract of Gymnema sylvestre showed anti-inflammatory activity in Wistar rats where carrageenan-induced inflammation was introduced in the rats. Methanolic extract of this plant reduced carrageenan-induced rat paw edema significantly (*p* < 0.05) (Kumar et al., 2012). In another study, aqueous extract of this plant displayed inhibitory potential against carrageenan-induced rat paw edema and peritoneal ascites in mice (Diwan et al., 1995). Furthermore, ethanolic extract of this plant was reported to show inhibitory effects against TPA-induced inflammation, with a 50% inhibitory dose of 50–555 nmol/ear where *In vivo* two-stage carcinogenesis was introduced in mice using 7,12-dimethylbenz[a]anthracene as an initiator and 12-O-tetradecanoyl phorbol-13-acetate (TPA) as a promoter (Yasukawa et al., 2014).

### Effect on Gastrointestinal Tract

Methanolic extract of Gymnema sylvestre showed anti-ulcer activity in pylorus ligated Wister rat, forced swim stress-induced ulcer model as well as in rats where ulcer was induced by Indomethacin. It reduced the ulcer index significantly (*p* < 0.01) and also reduced free acidity, total acidity and gastric volume, and increased the pH of gastric juice. It was suggested that anti-ulcer activity was due to the presence of phytochemical constituents such as saponins, flavanoids, tannins, sterols, glycosides, alkaloids, resins, carbohydrates, proteins, triterpenoids (Swetha et al., 2012). A herbomineral formulation containing *Gymnema sylvestre* was found to improve impaired gastric emptying and intestinal transit associated with diabetes. In this study, *Gymnema sylvestre* containing formulation was found to significantly restore gastric emptying time and intestinal transit (*p* < 0.001 and *p* < 0.001 respectively), (Somani et al., 2013). However, *Gymnema sylvestre* was also shown to inhibit Glucose-Stimulated Gastric Inhibitory Peptide Secretion in Wister Rats significantly (*p* < 0.05) (Fushiki et al., 1992). Ethanolic extract of the leaves was described to improve mucosal injury induced by ethanol in Wister Albino rats. In this study it was observed that treatment of rats with this plant extract resulted in a significant depletion of stomach-wall mucus (*p* < 0.001), total proteins (*p* < 0.01), nucleic acids (*p* < 0.001), and non-protein sulfhydryl groups (*p* < 0.001) (Al-Rejaie et al., 2012). In a study, where gastric ulcer was induced in Swiss Albino male mice, aqueous extract of this plant was reported to show anti-ulcerative properties where it was observed that treatment with the plant extract exhibited significant (*p* < 0.05) protective activity against aspirin induced ulcer in rat models (Arun et al., 2014).

### Hepatoprotective Activity

In an in-vitro investigation, hydro-alcoholic extract of *Gymnema sylvestre* was observed to render anti-hepatotoxic function in a dose-dependent manner in isolated rat hepatocytes where hepatotoxicity was induced using D – galactosamine. A significant increase in the level of ASAT, ALAT, ALP, total bilirubin and direct bilirubin (*p* < 0.001) was observed (Srividya et al., 2010). It was reported to lower urea and creatinine levels after acute and chronic administration of methanolic extract of this plant in Wister rats (Dholi and Raparla, 2014). In a study where methanolic poly herbal preparation containing this plant was used, it was observed that the preparation can reverse hepatotoxicity in Albino rats induced by paraffin and carbon tetrachloride (Yogi and Mishra, 2016).

### Effect on Body Weight

Ethanol extract of this plant was found to reduce body weight in Wister rats (Shigematsu et al., 2001a). Similarly, when Streptozotocin-induced diabetic Albino rats were treated with ethanolic extract of Gymnema syvestre, a significant (*p* < 0.001) weight reduction was also observed (Fatani et al., 2015). However, in a different study, ethanol extract of this plant was reported to cause increase in the weight of the whole body, liver, pancreas in alloxan induced diabetic Wistar rats (Ahmed et al., 2010).

### Anticaries Activity

The methanol extract of *Gymnema sylvestre* disclosed antimicrobial activity against *Streptococcus* mutans which is responsible for the formation of dental caries (Devi and Ramasubramaniaraja, 2010). Gymnema Acid from this plant can reduce glucan as well as plaque formation by *Streptococcus mutants* (Porchezhian and Dobriyal, 2003).

## Clinical Study Reports of *Gymnema Sylvestre*

Apart from various investigations on animal models, different extracts of this plant were tested on humans to inspect its therapeutic potential on the human body. Clinical investigations on *Gymnema sylvestre* revealed its potential to reduce body weight and glucose levels, triglyceride, LDL-c, total cholesterol and elevate the amount of insulin and C-peptide available in blood. A study conducted on 58 patients with type 2 diabetes mellitus for 90 days resulted in the reduction of fasting (p < 0.005) and post prandial blood glucose levels (p < 0.001) along with a reduction of triglyceride (*p* < 0.05) (**Table 4**) (Kumar et al., 2010). In another study, where 64 individuals with type 1 diabetes were treated with Gymnema leaf extract for 6 to 30 months resulted with the reduction of plasma glucose level, reduced external insulin dose and significant reduction in HbA1c (*p* < 0.001) (Shanmugasundaram et al., 1990b). Significant (*p* < 0.05) reduction in blood glucose level was observed in another study where 32 human subjects with type-2 diabetes were administered with *Gymnema sylvestre* leaf powder in hard gelatin capsule for 30 days. Reduction in triglyceride, cholesterol and LDL level was also observed in this study (Li et al., 2015) (**Table 4**). The therapeutic potential of *Gymnema sylvestre* (GS) observed from clinical studies so far conducted has been summarized in **Table 4**.

**Table 4:** Summary of clinical study results on *Gymnema sylvestre*.

Preparation of the plant given	Number of subjects	Duration	Therapeutic potential	Reference
OSA (Novel high molecular weight GS preparation)	11	60 days	• Significant reduction in fasting and post-prandial blood glucose level (*p* < 0.005 and *p* < 0.02 respectively) • Significantly increased level of insulin and C-peptide in blood (*p* < 0.05 and *p* < 0.001, respectively)	(Al-Romaiyan et al., 2010)
Extract of leaves in capsule form	58 human subjects with type-2 diabetes	90 days	• Reduction in hyperglycemia and hypertriglyceridemia (*p* < 0.05) • Reduced fasting and post-prandial blood glucose levels significantly (*p* < 0.005 and *p* < 0.001, respectively) • Significantly increased HbA1c level ( *p* < 0.001) • Significantly reduced insulin resistance (*p*< 0.05)	(Kumar et al., 2010)
GS leaf powder in hard gelatin capsule	32 human subjects with type-2 diabetes	30 days	• Significantly reduced glucose level (*p* < 0.05) • Reduced triglyceride, cholesterol and LDL level	(Li et al., 2015)
Aqueous GS leaf extract	8 healthy participants	90 min	• Reduced oral sweet taste sensation significantly (*p* < 0.05) • Reduced blood glucose level	(Kashima et al., 2017)
Ethanol extract of GS leaves	22 type 2 diabetic patient	18-20 months	• Reduced glucose level significantly (*p* < 0.001) • Elevated serum insulin in both the fasting and post-prandial state	(Baskaran et al., 1990)
Leaf extract of GS	64 individuals with type 1 diabetes	6 to 30 months	• HbA1c level was reduced significantly (*p* < 0.001) • Reduced glucose level • Reduced requirement of Insulin	(Shanmugasundaram et al., 1990b)
*G. sylvestre* (Swanson Premium *G*. *sylvestre* leaf; Swanson Health Products, Fargo, ND, USA)	24 diabetic patients	12 weeks	• Reduction in body weight, body mass index (BMI) significantly (*p* < 0.05 and *p* < 0.05, respectively) • Decreased level of very low-density lipoprotein (VLDL) significantly (*p* < 0.05)	(Zuñiga et al., 2017)
Calcium-potassium salt of (-)-hydroxycitric acid, niacin-bound chromium and GS	90 obese subjects	8 weeks	• Reduced weight	(Preuss et al., 2005)
Drops of GS “Q” with 1/4 cup of water	21 people type 2 diabetes	6 months	• Controlled blood glucose level	(Kothe and Uppal, 1997)
Aqueous decoction of shade-dried powdered leaves of GS	10 healthy and 6 diabetic adults	10 days	• Reduced blood glucose level	(Khare et al., 1983)

## Toxicological Reports on *Gymnema Sylvestre*

Toxicology study on Albino mice treated with Gymnema sylvestre showed LD50 level at 3990 mg/kg and the safety ratio for normal and diabetic mice was found to be 11.08 and 16.03 respectively. In this study, no behavioral, neurologic and autonomic adverse effects were observed (Chattopadhyay, 1999). Another study reported LD50 of ethanol and water extract of *Gymnema sylvestre* to be 375mg/kg where mice were treated by intraperitoneal route with the plant extract (Bhakuni and Dhar, 1971). One case of drug-induced liver injury (DILI) was informed in the case of a patient who was treated with *Gymnema sylvestre* for diabetes mellitus (Shiyovich et al., 2010). It was stated that this plant can cause hypoglycemia in both diabetic and non-diabetic patient (Khare et al., 1983) and in the case of the diabetic animal model, one study reported persistent hypoglycemic effect even after the treatment with Gymnema sylvestre was stopped (Srivastava et al., 1986). However, no toxic effects were observed in a study where male and female Wistar rats were treated with *Gymnema sylvestre* for 52 weeks (Ogawa et al., 2004).

## Use of *Gymnema Sylvestre* as Dietary Supplement

Concerning the use of extract of *Gymnema sylvestre* as a dietary supplement in Europe, the European Food Safety Agency recognizes the property of this plant which maintains normal sugar levels in organisms and in their conditions of use it must contain 400- 800 mg of gymnema extract, equal to 100- 200 mg kgymnemic acid (EFSA Panel on Dietetic Products, Nutrition and Allergies (NDA), 2010).

Presently, there are considerable knowledge gaps for the risk assessment of *G. sylvestre* preparations and open questions for whether results obtained with one preparation can be extrapolated to another Gymnema preparation. Also, based on the lack of systematic data on dose and effect relationships, the available information was regarded as not being sufficient for the derivation of health-based guidance values for Gymnema or Gymnema preparations. Considering the uncertainties for the composition of different Gymnema preparations, potential herb–drug interactions and the concerns about glucose lowering or hypoglycaemic effects, the use of Gymnema-based food supplements in combination with (or as a substitute for) authorized antidiabetic drugs may be associated with risks when used without medical supervision (Marakis et al., 2018).

## Summary and Future Perspectives

Phytochemicals account for numerous pharmacological properties. They are observed to have anti-metastatic, anti-diabetic, hypoglycemic, anti-oxidant, hepatoprotective, anti-inflammation, anti-bacterial, anti-fungal, anti-viral etc. activities. Plants contain compounds such as flavonoids, alkaloids, and tannins that render these life-saving therapeutic activities. It has been reported that about 80% of people from developing countries rely on natural medicines for the treatment of diseases and their primary health concerns (Hamilton, 2004). However, despite having great demand and therapeutic uses only 10% of the plants have been investigated for their therapeutic potential (Kumar V.H, et al., 2015). Furthermore, some of these plants which could be a great source of biologically important novel phytoconstituents are on the verge of extinction due to unsustainable use, destruction of forests, and habitat destruction (Brower, 2008). One of these therapeutically important plants that contain significant biologically important phytochemicals is *Gymnema sylvestre*. It constitutes saponins, flavonol, glycosides, gymnemanol, gurmarin etc. These phytochemicals isolated from *Gymnema sylvestre* can provide pharmacological activities such as anti-diabetic, anti-oxidative, anti-metastatic, anti-inflammatory, lipid-lowering and several other properties.

However, this plant is also subject to unsustainable use. It is disappearing very fast due to overexploitation and extensive collection to meet the demand (Choudhury, 1988). Many unauthorized preparations of this plant are found in the local market. People are using this plant as a cheap substitution for their anti-diabetic medicine without any knowledge of what part of the plant to be used which results in unnecessary destruction the whole plant. Thus, this plant is being wasted without being used up to their maximum potential. In order to prevent the waste of this plant, legal production of medicinal preparation from the plant should be ensured and sustainable use of this plant should be closely monitored. In addition to these, people should be also made aware of the proper use of the plant so that they can get maximum benefit from this plant.

## Author Contributions

MMRS conceived the concept. FK and MMRS wrote the initial draft and revised the manuscript. LCM, INM, CZ and MAR critically revised the manuscript. BYS and HFT significantly contributed to review the manuscript in reply to reviewers; FK, MMRS, LCM, INM, CZ, BYS, HFT and MAR finalized the manuscript. All authors read and approved the manuscript.

## Conflict of Interest

The authors declare that the research was conducted in the absence of any commercial or financial relationships that could be construed as a potential conflict of interest.
